# Global Transcriptome Analysis of Combined Abiotic Stress Signaling Genes Unravels Key Players in *Oryza sativa* L.: An *In silico* Approach

**DOI:** 10.3389/fpls.2017.00759

**Published:** 2017-05-15

**Authors:** Pandiyan Muthuramalingam, Subramanian R. Krishnan, Ramanujam Pothiraj, Manikandan Ramesh

**Affiliations:** Department of Biotechnology, Alagappa UniversityKaraikudi, India

**Keywords:** abiotic stress genes, *Oryza sativa*, stress signaling, comparative mapping, protein–protein interactions, combined abiotic stress, INMEX

## Abstract

Combined abiotic stress (CAbS) affects the field grown plants simultaneously. The multigenic and quantitative nature of uncontrollable abiotic stresses complicates the process of understanding the stress response by plants. Considering this, we analyzed the CAbS response of C3 model plant, *Oryza sativa* by meta-analysis. The datasets of commonly expressed genes by drought, salinity, submergence, metal, natural expression, biotic, and abiotic stresses were data mined through publically accessible transcriptomic abiotic stress (AbS) responsive datasets. Of which 1,175, 12,821, and 42,877 genes were commonly expressed in meta differential, individual differential, and unchanged expressions respectively. Highly regulated 100 differentially expressed AbS genes were derived through integrative meta-analysis of expression data (INMEX). Of this 30 genes were identified from AbS gene families through expression atlas that were computationally analyzed for their physicochemical properties. All AbS genes were physically mapped against *O. sativa* genome. Comparative mapping of these genes demonstrated the orthologous relationship with related C4 panicoid genome. *In silico* expression analysis of these genes showed differential expression patterns in different developmental tissues. Protein–protein interaction of these genes, represented the complexity of AbS. Computational expression profiling of candidate genes in response to multiple stresses suggested the putative involvement of OS05G0350900, OS02G0612700, OS05G0104200, OS03G0596200, OS12G0225900, OS07G0152000, OS08G0119500, OS06G0594700, and Os01g0393100 in CAbS. These potential candidate genes need to be studied further to decipher their functional roles in AbS dynamics.

## Introduction

Plants, in particular cereal food crops play a major role in survival of human beings. Unlike animals, plants are sessile and hence, cannot escape stressful conditions, that originate from the abiotic stresses (drought, salinity, metal, and submergence), which may lead to the loss in yield and nutritional value (Rizhsky et al., 2002, 2004; Mittler, 2006; Mittler and Blumwald, 2010; Atkinson and Urwin, 2012). Each abiotic stressors affects the plants at different levels, heat stress reduces the yield of the plants and drought affects its growth (Rizhsky et al., 2002, 2004; Mittler, 2006; Acquaah, 2007; Thakur et al., 2010; Prasad et al., 2011; Vile et al., 2012). The effect of an individual stressor on the plant may modulate its susceptibility to the concurrently combined multiple stresses. Previous studies have shown that water deficit or avoidance to salinity, may increase the susceptibility of plant to biotic stress (Siddiqui, 1980; Xu et al., 2008). Plants are highly developed from all the surrounding defense responses by acclimatizing to CAbS (Mickelbart et al., 2015). The exertion of combined stresses triggers the activation of several ion channels leading to the accumulation of hormonal changes, which in turn increases the production of reactive oxygen species (ROS). ROS acts as signaling molecules and are also expected to cause cellular damages (Mittler, 2002; Apel and Hirt, 2004; Kissoudis et al., 2014). Upon exposure to stress conditions, non-specific signals are transduced, which in turn generate physiological, molecular and metabolic responses that eventually alters the stress tolerance (Rizhsky et al., 2004; Koussevitzky et al., 2008; Atkinson et al., 2013; Iyer et al., 2013; Prasch and Sonnewald, 2013; Rasmussen et al., 2013; Lata et al., 2015; Ramegowda and Senthil-kumar, 2015). Thus, the overall effect of a stress combination may be entirely different from the individual stresses, which makes it appropriate to analyze the effect of a combination of stress by subjecting them to a CAbS (Mittler, 2006; Atkinson and Urwin, 2012; Prasch and Sonnewald, 2013). How plants distinguish the CAbS leading to gene regulation is far from clear. Global agricultural production will face abounding challenges over the coming decades, mainly due to climate change. It will be a tough task for the farmers to increase the production under adverse climatic conditions, where combination of stresses will play a dominant role (Munns and Tester, 2008; FAO, 2009; Zhao and Running, 2010; Peters et al., 2011; Shanker and Venkateswarlu, 2011; Kissoudis et al., 2014). So understanding the molecular responses to these combined stresses in crop plants are unconditionally needed for the future food security. Apart to literary survey, huge amount of transcriptomic data are available in the field of abiotic stress biology. Comparison of the transcriptomic data of plants exposed to the individual and combined stresses is expected to reveal the importance of the molecular cross-talks specific to a particular stress or CAbS. The available transcriptomic information can thus be utilized to understand responses of plants to the combined stress (Ma et al., 2007; Narsai et al., 2013a). Easily accessible meta-analysis data are vital for this direction. *Arabidopsis thaliana* and *O. sativa* subjected to bacterial and water deficit stresses resulted in a number of commonly regulated stress-responsive genes by meta- analysis of microarray data (Shaik and Ramakrishna, 2013, 2014). To our knowledge, this is the first time an effort was made to compare the transcriptomic data of rice plants for understanding the molecular mechanisms involved in specific and CAbS responses. Herein, the comprehensive meta-analysis was performed on rice transcriptome datasets selected from 8 freely accessible abiotic stress experiments to identify commonly regulated genes. We predicted that 30 AbS responsive genes were expressed across the CAbS. In addition, similar computational analysis of these genes confirmed their expression pattern observed in the microarray. This analysis led to the identification of unique and combined sets of AbS genes for further characterization toward outlining their functional role of AbS signaling in rice.

## Materials and methods

**Figure 1:**
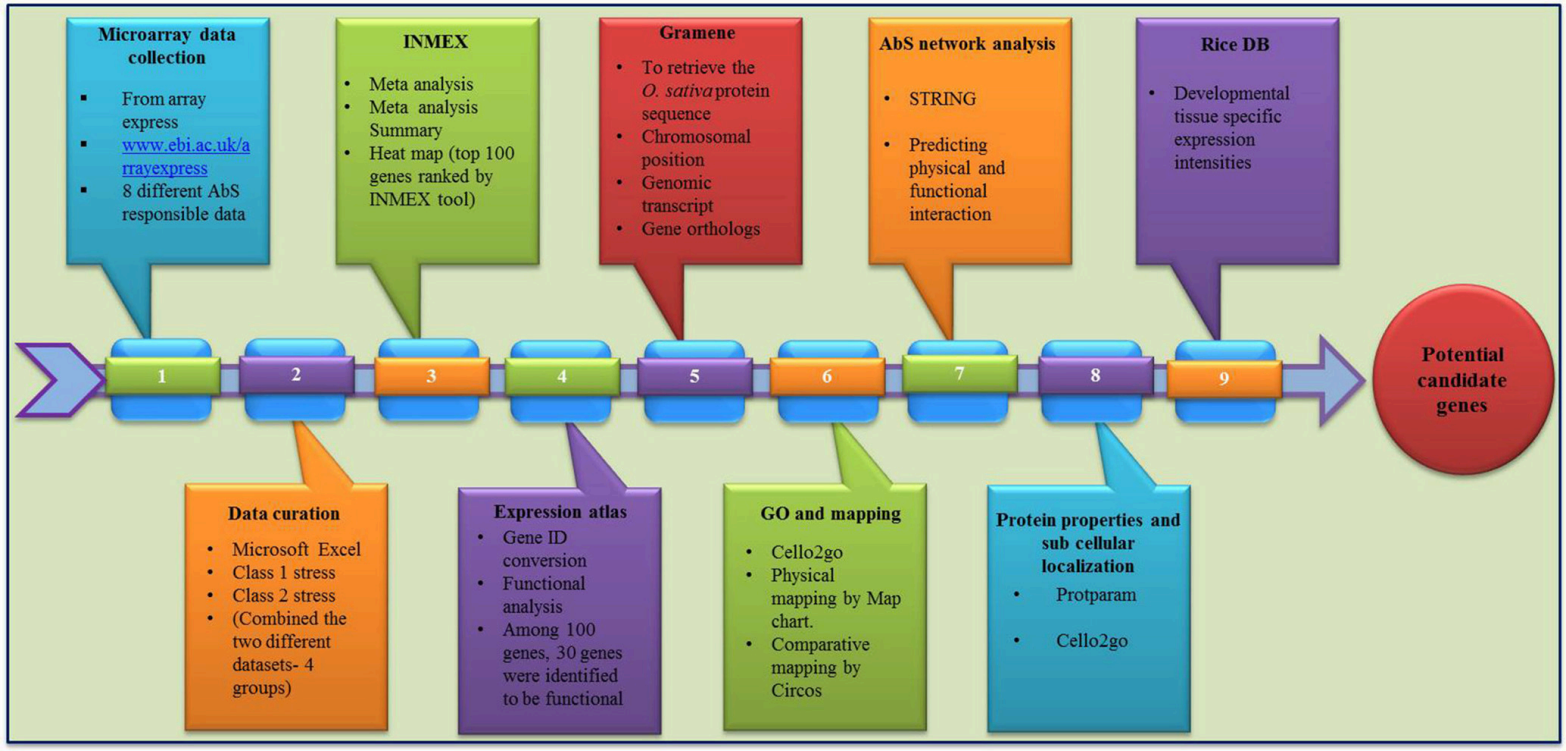
**Overall work flow**.

The overall frame work from data mining followed by grouping and analyzing the CAbS responsive genes of this transcriptomic study is illustrated in framework Figure 1.

### Computational mining of CAbS responsive genes from rice transcriptome

The global transcriptomic data of individual abiotic stress on rice (*O. sativa*) were collected from Array Express database (https://www.ebi.ac.uk/arrayexpress/; Parkinson et al., 2007). Collected data sets were curated manually using MS Excel and signalized as drought, salinity, submergence, metal, natural expression, biotic and abiotic stress were data mined through publically accessible transcriptomic AbS responsive datasets (Table 1). Further these AbS transcriptomic data were compiled into four groups and then imported to INMEX tool (http://www.inmex.ca/; Xia et al., 2013). Stouffer's model was used to identify the commonly-shared genes and the AbS datasets were integrated revealing the AbS genes functioning under different stress conditions. The data sets were uploaded using input file format (.txt or .zip) that was plotted in Excel file with gene expression values, corresponding probe ID or samples or experiments in columns and rows with gene name. Each column or treatments were named as per specific treatment. Further the up and down regulated gene IDs were converted from probeset ID to Ensembl IDs by Expression Atlas (https://www.ebi.ac.uk/gxa/about.html; Petryszak et al., 2016). The Abiotic stress responsive gene IDs (AbS responsive gene IDs) were used to retrieve the protein sequence from Gramene (Tello-Ruiz et al., 2016). The identified abiotic stress protein sequences were searched using BLASTP against *O. sativa* of Gramene to retrieve corresponding genomic transcripts and coding sequences with their chromosomal positions.

**Table 1:** **Name and technical details of transcriptome data**.

**S. no**	**Name of array**	**Technical details of array**
1	E-GEOD-62308	Expression data from ABA up-regulated rice under drought stress
2	E-GEOD-58603	Genome wide gene expression profiling of salinity responsiveness
3	E-GEOD-41647	Transcriptome profiling for drought tolerant and susceptible cultivars of *indica* rice
4	E-GEOD-41103	Rice submergence stress response
5	E-GEOD-21651	Differential expression of salt and drought stress
6	E-GEOD-41733	Chromium stress response in rice roots: Effects on transcriptome profiles and signaling pathway response
7	E-GEOD-38102	Expression data from *Oryza sativa* and *Arabidopsis thaliana*
8	E-GEOD-51616	*Ghd7* is a central regulator for growth, development, adaptation and responses to biotic and abiotic stresses

### Gene ontology (GO) annotation

Gene identities corresponding to 30 different AbS responsive genes were loaded into the CELLO2GO (http://cello.life.nctu.edu.tw/cello2go/; Yu et al., 2014) to obtain the GO annotation against eukaryote. Genes were also categorized as per GO biological process, its molecular function according to CELLO2GO GO functional classification.

### Identification and prediction of unique and combined AbS responsive genes

AbS responsive genes from combined stress were compared using Venn diagrams (http://bioinfogp.cnb.csic.es/tools/venny/; Oliveros, 2015). Based on Venn intersections, unique and combined AbS genes were segregated based on the transcriptomes and 30 AbS responsive genes as common stressed rice transcriptome.

### Prediction of chromosomal location

The individual and CAbS responsible genes were searched against the *O. sativa* ssp. *japonica* genome in Gramene. Chromosomal locations including the chromosome number, position of gene were also identified. The genes were then plotted individually onto the respective rice chromosomes as per their ascending order of physical position (bp), from the short arm telomere to the long arm telomere and the resultant physical maps were displayed using MapChart v2.3 (Voorrips, 2002).

### Protein feature analysis

The physicochemical properties including molecular weight, isoelectric point (pI), number of positive and negatively charged residues and the instability index were predicted using protparam tool of ExPASy (Gasteiger et al., 2005).

### Analysis of subcellular localization in AbS proteins

The subcellular localizations of unique and combined AbS responsive proteins of *O. sativa* were predicted using CELLO2GO (http://cello.life.nctu.edu.tw/cello2go/; Yu et al., 2014).

### Comparative mapping of rice AbS responsive genes in related grass genome

The amino acid sequences of physically mapped unique and CAbS genes were BLASTP searched against the peptide sequences of sorghum, maize, foxtail millet (http://gramene.org/) to predict the respective orthologs in these grass species. All hits with at least 60% homology were considered significant. The orthologous relationships between rice and these grass species were then visualized using Circos v0.55 (http://circos.ca/; Krzywinski et al., 2009).

### Signal transduction network analysis

To facilitate the usage of these annotated AbS genes (Ensembl Gene IDs) that is exposed to STRING tool for predicting the gene interaction at physical and functional level (Szklarczyk et al., 2015).

### Developmental tissue-specific expression analysis

The unique and combined AbS responsive genes with their respective gene IDs were subjected to Rice DB (http://ricedb.plantenergy.uwa.edu.au; Narsai et al., 2013b). Developmental tissue-specific expression with its regulations, functions and evolutionary informations were analyzed based on the data retrieved.

## Results

### CAbS genes from meta-analysis of rice transcriptome

**Figure 2:**
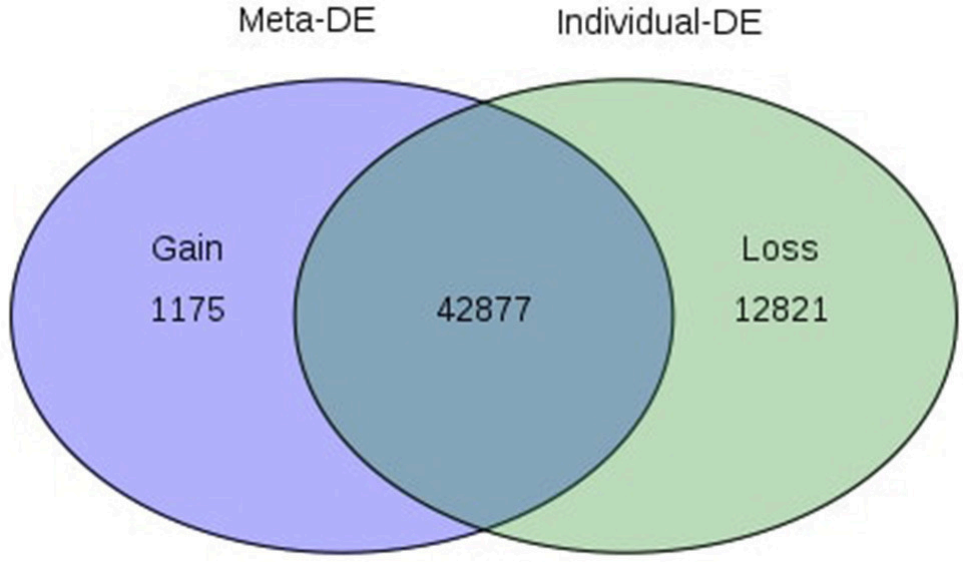
**Venn diagram depicting commonly expressed genes from CAbS**. DE, Differential expression. The processed data were integrated in meta-analysis tool and differentially expressed CAbS genes were identified.

**Figure 3:**
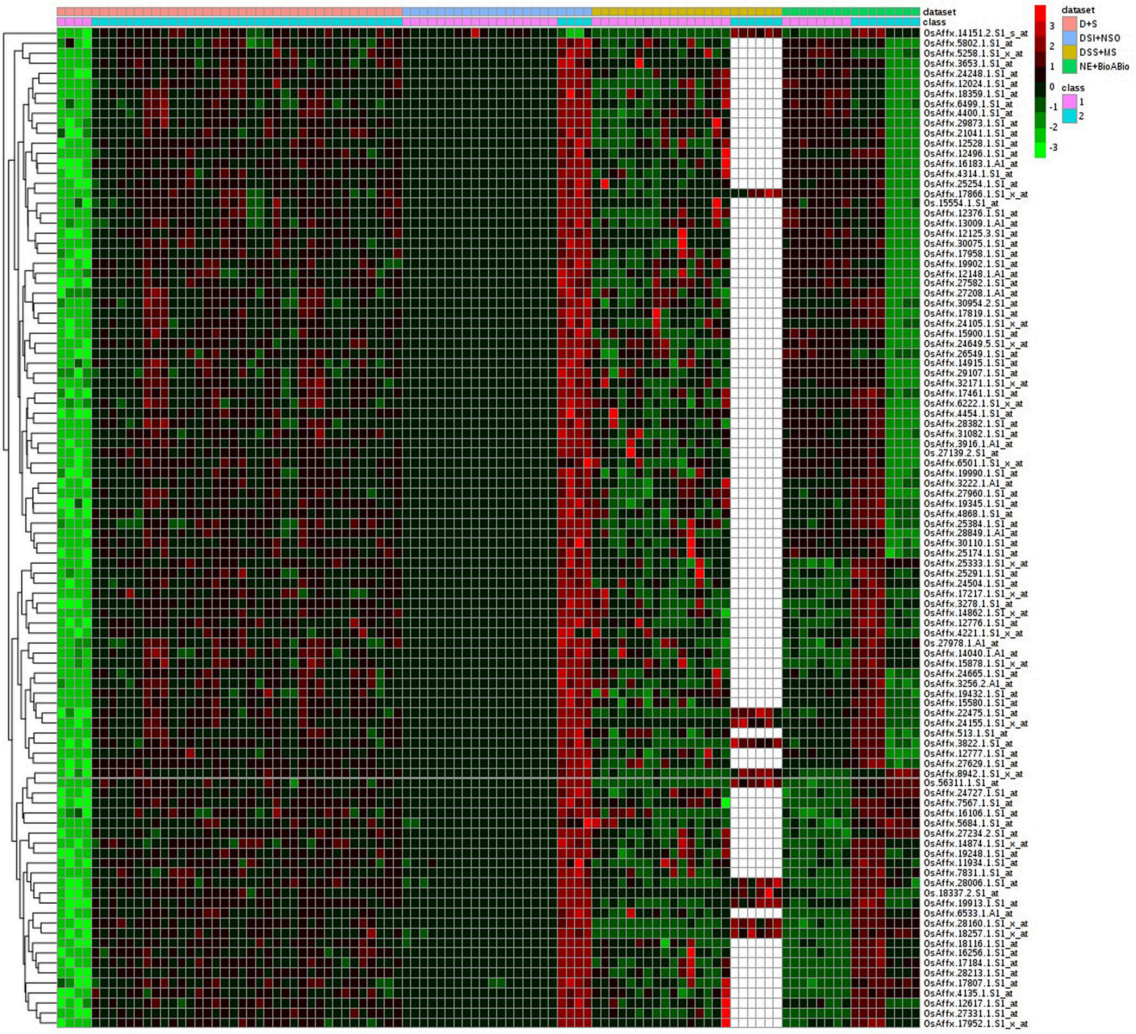
**Heatmap representing the top 100 genes which are differentially expressed across CAbS**. Red color, up regulation; Green color, down regulation; Black color, unchanged; White color, missing data values. Datasets: Pink color, D+S; Blue color, DSI+NSO; Olive color, DSS+M; Green color, NE+BioABio are used. (D+S, Drought+Salinity; DSI+NSO, Drought stress *indica* + Natural stress *Oryza*; DSS+M, Drought, Salinity Stress+Metal; NE+BioABio, Natural Expression+Biotic and Abiotic expression). Class 1- D, DSI, DSS, NE; Class 2- S, NSO, MS, BioABio. Class 1 and 2 present in 4 different AbS responsible datasets.

**Figure 4:**
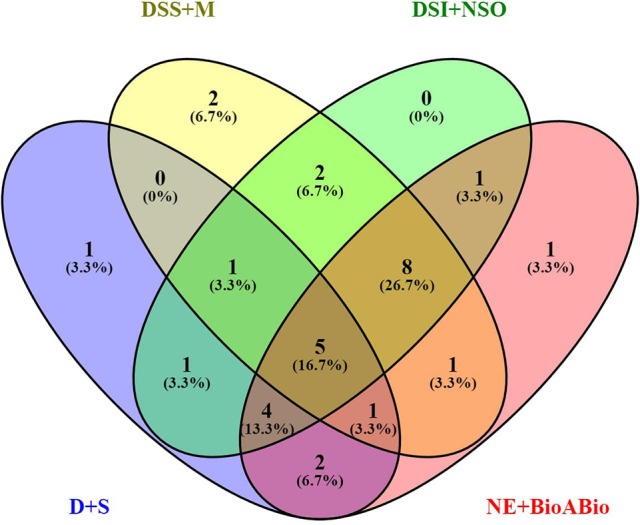
**Schematic representation of CAbS responsive genes**. DSS+M (Drought, salinity stress + Metal); D+S (Drought + Salinity); NE+BioABio (Natural expression + Biotic and Abiotic); DSI+NSO (Drought stress *indica* + Natural stress *Oryza*). Venn diagram indicates combinatorial stress study found unique and CAbS stress responsible genes.

Of the 8 different AbS datasets compiled, 1,175, 12,821, and 42,877 genes were commonly expressed in meta differential, individual differential, and unchanged expressions respectively (Table 1 and Figure 2). Hundred differentially expressed genes with respect to abiotic stress were predicted by heatmap (Figure 3) of which 30 unique and CAbS genes were predicted by expression atlas. The Figure 4 depicts 4 possible combinatorial studies and found 30 common genes which are differentially regulated. Then Gramene search for abiotic stress proteins in *O. sativa* ssp. *japonica* showed the presence of 29 out of 30 unique and CAbS proteins. Among these, one AbS responsive protein (Os06G0594600) was found to be an alternate transcript (Table 2).

**Table 2:** **AbS responsive gene description**.

**S. no**	**Designing element**	**Ensemble ID**	**Gene description**
1	OsAffx.14151.2.S1_s_at	OS04G0433000	TRAF like domain
2	OsAffx.12528.1.S1_at	OS02G0695050	Protein of unknown function DUF868
3	OsAffx.4314.1.S1_at	OS05G0215183	Putative uncharacterized protein
4	OsAffx.17866.1.S1_x_at	OS09G0403300	Cytochrome P450 family
5	OsAffx.24649.5.S1_x_at	OS02G0604800	Putative cyclin–F1–3
6	OsAffx.14915.1.S1_at	OS05G0390000	Fungal lipase like domain, alpha /beta hydrolase fold domain
7	OsAffx.32171.1.S1_x_at	OS01G0220900	Putative uncharacterized protein
8	OsAffx.4454.1.S1_at	OS05G0350900	Homeodomain, MYB domain
9	Os.27139.2.S1_at	OS02G0612700	BTB domain, potassium channel tetramerization
10	OsAffx.6501.1.S1_x_at	OS09G0543400	Ornithine/DAP/Arg decarboxylase family
11	OsAffx.19345.1.S1_at	OS11G0650600	Von willebrand factor type A
12	OsAffx.28849.1.S1_at	OS07G0600300	APO domain
13	OsAffx.14862.1.S1_x_at	OS05G0353600	Glutaredoxin and thioredoxin like fold
14	OsAffx.12776.1.S1_at	OS03G0179100	Germin, Cupin 1, Rml C like cupin domain
15	OsAffx.4221.1.S1_x_at	OS05G0104200	Leucine rich repeat
16	OsAffx.24155.1.S1_x_at	OS02G0174400	Protein Kinase domain
17	OsAffx.3822.1.S1_at	OS04G0255600	Cytochrome P450 Conserved site
18	Os.56311.1.S1_at	OS03G0596200	Zinc finger CCHC like domain
19	OsAffx.7567.1.S1_at	OS12G0225900	Alcohol dehydrogenase super family and chaperonin 10 like domain
20	OsAffx.16106.1.S1_at	OS07G0152000	Transcription factor and TCP subgroup
21	OsAffx.5684.1.S1_at	OS08G0119500	Methyltransferase 11 like domain
22	OsAffx.14874.1.S1_x_at	OS05G0360900	Pectinesterase inhibitor domain
23	OsAffx.19248.1.S1_at	OS11G0605200	Putative uncharacterized protein
24	OsAffx.7831.1.S1_at	OS12G0572700	Exostosin family
25	OsAffx.28006.1.S1_at	OS06G0594700	Transferase, chloramphenicol acetyltransferase like domain
26	Os.18337.2.S1_x_at	OS01G0393100	NAC domain
27	OsAffx.18257.1.S1_x_at	OS10G0211900	Putative uncharacterized protein
28	OsAffx.16256.1.S1_at	OS07G0244800	Pectinesterase inhibitor domain
29	OsAffx.17807.1.S1_at	OS09G0375300	PMR5- N terminal doamin
30	OsAffx.3256.1.S1_at	OS03G0278566	Putative uncharacterized protein

### Functional GO annotation

**Figure 5:**
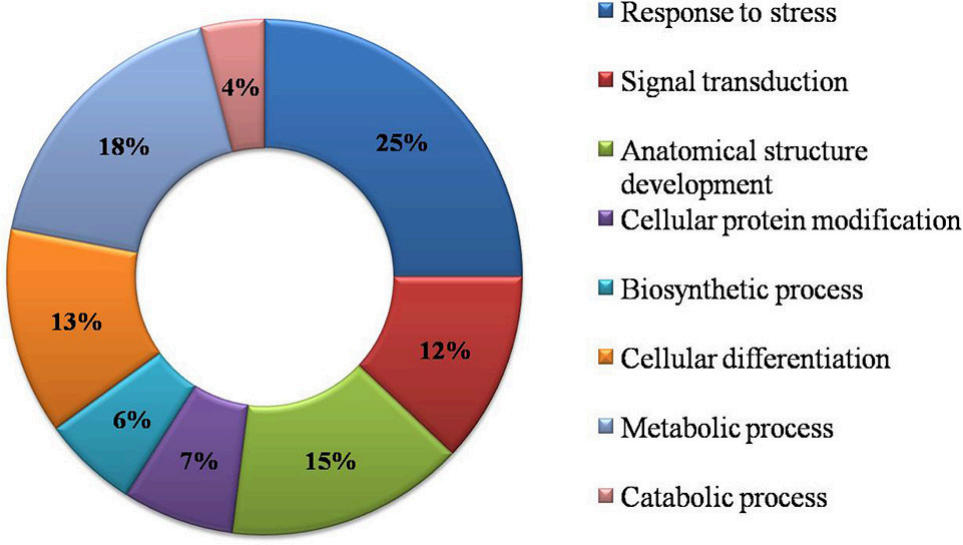
**Classification of CAbS based on their biological process**. GO biological process based categorization for differentially expressed genes (DEGs) unique to combined stresses.

**Figure 6:**
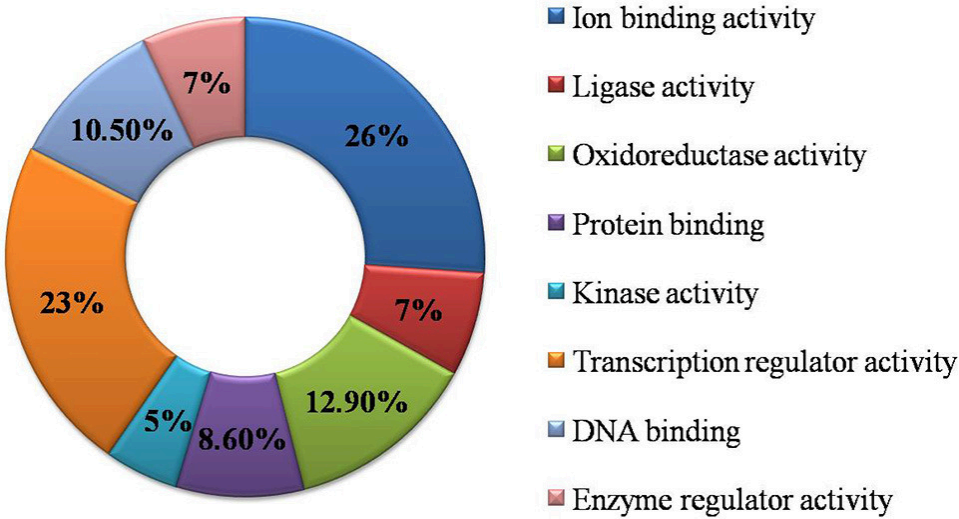
**Classification of commonly expressed genes according to their molecular function**. GO molecular function based categorization for DEGs unique to combined stresses.

Functional GO annotation of 30 CAbS proteins predicted the involvement of these proteins in various molecular functions and biological processes (Supplementary Table 1). A majority of unique and CAbS proteins were predicted to be involved in response to stress, cellular protein modification, cell differentiation, signal transduction, anatomical structure development, metabolic, and biosynthetic processes (Figure 5). Furthermore molecular functions of these proteins corresponded to ion binding, transcription regulation and oxidoreductase activity (Figure 6).

### Developmental tissue-specific expression profiles of commonly shared AbS genes

Of the 30 unique and CAbS responsive genes, 28 genes showed tissue–specific expression pattern on various (41) plant developmental tissues. Few selected predominant genes OS05G0350900, OS02G0612700, OS05G0104200, OS03G0596200, OS12G0225900, OS07G0152000, OS08G0119500, OS06G0594700, and Os01g0393100 showed a higher expression in different developmental tissues like seed (S1 to S5), flower, seedling (1–4 week old seedlings), germination, leaf, and root. The genes showed a negligible expression in ovary, meristem, root, embryo, and endosperm as predicted by Rice DB based on the available AbS transcriptomic data (Supplementary Tables 3–11). AbS genes with annotations, main probesets, model sequences, motifs, and functional details are provided in Supplementary Table 2.

### Physical mapping of AbS responsive genes

**Figure 7:**
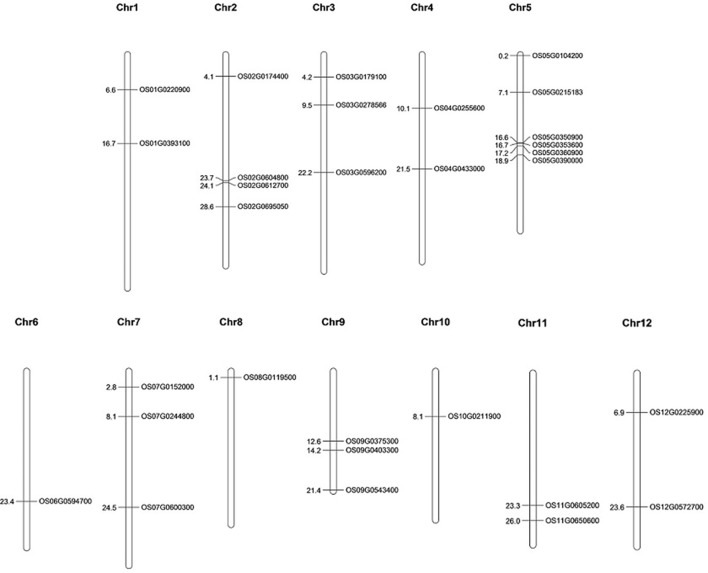
**Physical map of CAbS genes showing their chromosomal localization**. Vertical bars indicate the chromosomes and numbers at left denotes the position of genes (in Mb) [Physical map construction based on IRGSP v.2005].

The selected 30 AbS genes were annotated based on the chromosomal location of AbS responsive genes on rice genome and the physical map was constructed by plotting the genes onto 12 chromosomes of *O. sativa* ssp. *japonica*. Six AbS genes were localized at chromosome 5, 4 genes on chromosome 2, followed by 2 genes each on chromosomes 3, 7, and 9. Chromosomes 1, 4, 11, and 12 had 2 genes each. A single locus of AbS genes on chromosomes 6, 8, and 10 was mapped (Figure 7).

### Comparative mapping in related grass species

**Figure 8:**
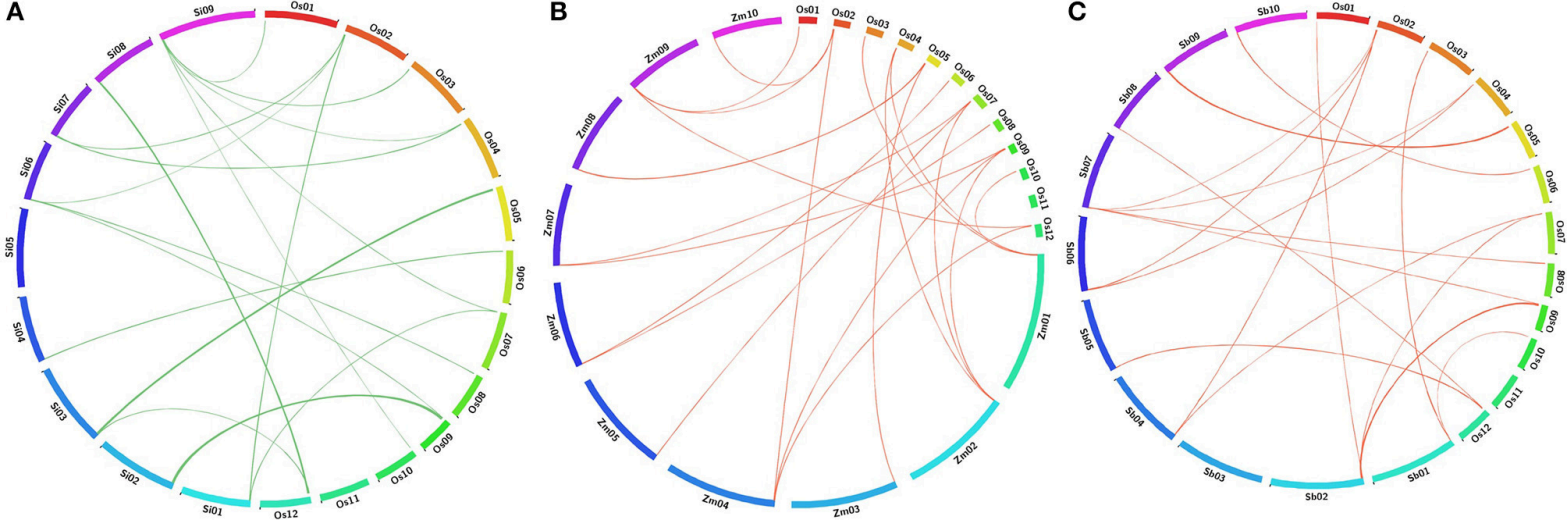
**Comparative map showing the orthologous gene of CAbS in *O. sativa* (Os) and (A)** *Setaria italica* (Si), **(B)** *Zea mays* (Zm), **(C)** *Sorghum bicolor* (Sb). Each segment indicates chromosome and the orthologous genomic regions are marked with red and green.

BLAST search analyses deduced the orthologous relationship among the 29 AbS responsive genes of rice (*japonica*) against related (C4) grass genomes of sorghum, maize, and foxtail millet by physical mapping and were compared with their respective chromosomes. The comparative mapping depicted maximum orthology between rice and foxtail millet [21 genes (~ 70%)], rice and maize [21 genes (~ 70%)], followed by rice and sorghum [20 genes (~ 67%)] (Figures 8A–C; Supplementary Tables 12–14).

### AbS gene interaction network

**Figure 9:**
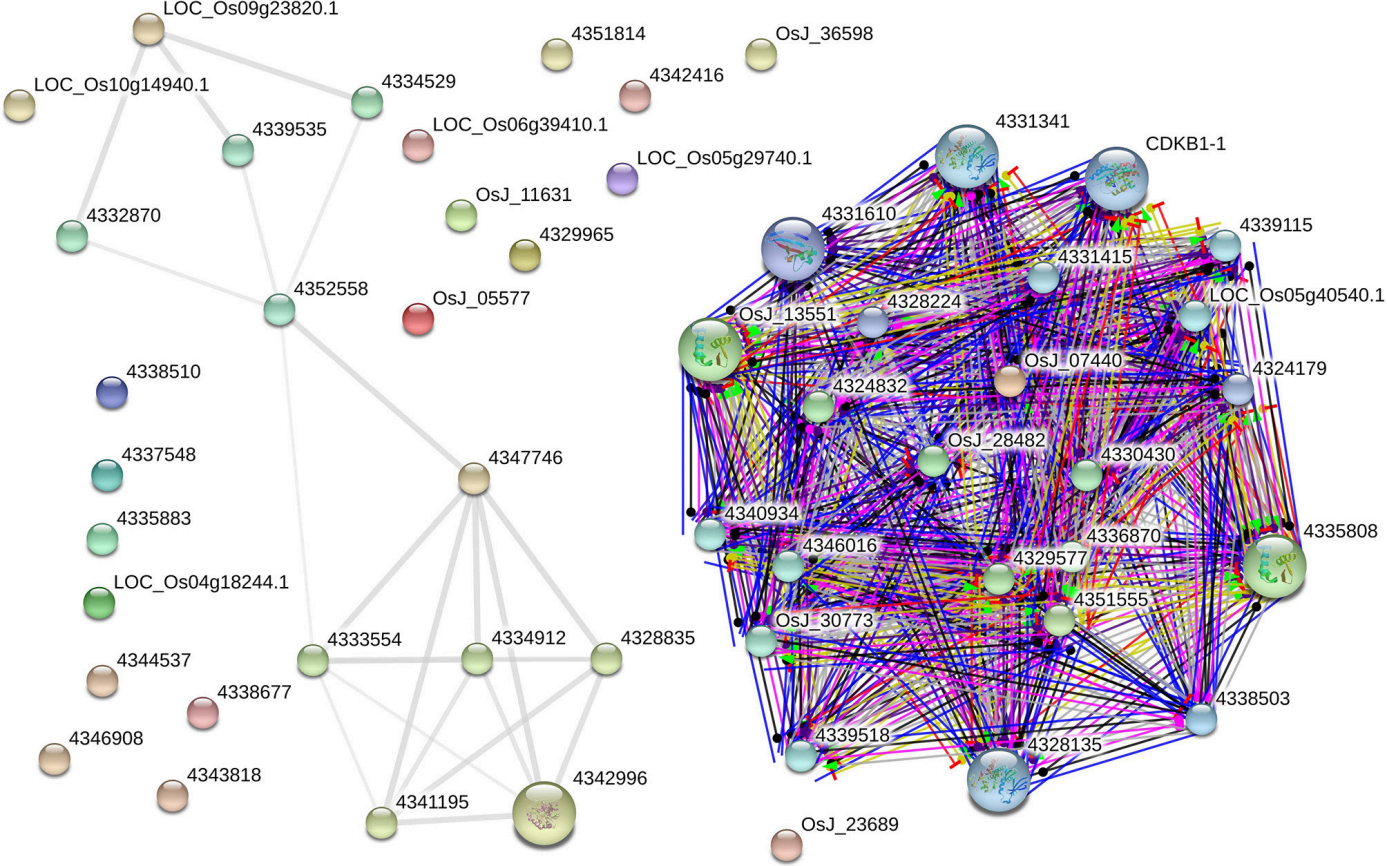
**AbS responsive genes interactome**. *Japonica* rice – AbS responsive gene interaction shows tightly connected functional modules. Colored line between the proteins which indicates various types of interaction evidence. Enlarged protein nodes that represent the availability of protein 3D structure information.

The AbS gene network from *O. sativa* ssp. *japonica* contains 22 proteins out of the 30 seed proteins and 34 neighbors were derived. Eighteen AbS responsive proteins had no links (i.e., there was no experimental evidence for interaction). Thus the network has 52 nodes and 201 edges (Figure 9). The seed - proteins of the AbS network have average node degree 7.73 than neighbor proteins. This molecular interaction revealed the complexity of AbS and thus proves multi–genic nature.

### Protein properties of commonly expressed AbS responsive genes

Among the 29 AbS responsive proteins, Os11G0605200 protein was the smallest with 94 amino acids and Os02G0174400 stood the biggest with 752 amino acids. With respect to protein lengths, molecular weights also varied accordingly with Os11G0605200 being the lowest (10.015 kD) and Os02G0174400 the highest (81.3 kD). Physiological stress genes were also predicted to have a diverse pI ranging from 4.36 (Os11G0605200) to 11.91 (Os09G0403300). Stability index of CAbS responsible genes indicates 20 unstable and 9 stable among 29 predicted proteins (Table 3). Added to it, aliphatic index and grand average of hydropathicity (GRAVY) were also determined. The aliphatic index was minimum for Os01G0393100 (53.84) and maximum for Os05G0215183 (104.48). The GRAVY values were minimum for Os04G0433000 (−0.946) and maximum for Os05G0360900 (0.341) (Table 3).

**Table 3:** **Protein properties and subcellular localization**.

**S. No**	**Gene ID**	**Protein length (aa)**	**Molecular weight**	**pI**	**Instability index**	**Stable or unstable**	**Aliphatic index**	**Grand average of hydropathicity (GRAVY)**	**SL**
1	OS04G0433000	344	37,261.5	11.29	56.58	Unstable	55.38	−0.946	N
2	OS02G0695050	260	29,399.8	11.25	51.11	Unstable	87.77	−0.356	Mt
3	OS05G0215183	134	14,287.1	4.85	56.37	Unstable	104.48	0.000	Ct
4	OS09G0403300	576	62,667.8	11.91	73.73	Unstable	73.47	−0.620	N
5	OS02G0604800	377	41,813.5	5.08	39.19	Stable	80.29	−0.185	PM
6	OS05G0390000	577	64,524.1	6.35	47.58	Unstable	73.97	−0.523	Mt
7	OS01G0220900	151	16,683.0	9.13	83.57	Unstable	66.62	−0.586	N
8	OS05G0350900	211	21,683.1	5.45	57.42	Unstable	69.57	−0.357	PM
9	OS02G0612700	458	49,196.1	5.97	33.58	Stable	75.48	−0.237	Cyto
10	OS09G0543400	428	45,418.9	5.96	28.52	Stable	86.96	0.088	Cyto
11	OS11G0650600	569	60,964.5	6.58	35.48	Stable	92.81	0.010	Cyto
12	OS07G0600300	418	45,788.2	8.57	45.16	Unstable	74.86	−0.468	Cyto
13	OS05G0353600	335	35,592.5	10.95	63.75	Unstable	66.21	−0.446	N
14	OS03G0179100	225	23,586.2	8.42	25.48	Stable	96.53	0.316	EC
15	OS03G0278566	123	12,898.7	11.58	61.51	Unstable	70.81	−0.433	N
16	OS02G0174400	752	81,372.3	11.54	74.92	Unstable	78.90	−0.524	N
17	OS04G0255600	543	59,264.7	6.48	49.23	Unstable	83.63	−0.165	Mt
18	OS03G0596200	382	42,581.2	10.04	60.50	Unstable	76.34	−0.452	N
19	OS12G0225900	345	37,963.8	6.96	22.28	Stable	90.90	0.013	Cyto
20	OS07G0152000	445	47,256.2	9.06	56.16	Unstable	62.00	−0.566	PM
21	OS08G0119500	122	13,463.3	7.16	50.62	Unstable	100.57	−0.002	Cyto
22	OS05G0360900	198	20,255.9	5.24	28.30	Stable	102.42	0.341	EC
23	OS11G0605200	94	10,015.3	4.36	25.33	Stable	103.83	0.120	Ct
24	OS12G0572700	526	58,563.4	9.51	55.96	Unstable	71.06	−0.400	PM
25	OS06G0594700	281	29,992.5	6.21	27.19	Stable	86.36	0.171	Ct
26	OS01G0393100	328	36,960.2	7.06	55.13	Unstable	53.84	−0.627	N
27	OS10G0211900	148	15,768.7	9.27	70.63	Unstable	65.95	−0.407	EC
28	OS07G0244800	204	21,656.6	9.07	46.64	Unstable	76.26	−0.218	EC
29	OS09G0375300	445	48,174.4	6.94	52.85	Unstable	71.15	−0.291	Ct

*aa, Amino acids; pI, Isoelectric point; SL, Subcellular localization; N, Nucleus; Mt, Mitochondria; Ct, Chloroplast; EC, Extra cellular; PM, Plasma membrane; Cyto, Cytoplasm*.

### Subcellular localization of AbS responsive proteins

Analysis of the subcellular localization of AbS responsive proteins in rice obtained an interesting pattern. It resembles 8 AbS responsive proteins in rice that were predicted to be localized in the nucleus, 4 in plasma membrane, extracellular, chloroplast each, 3 in the mitochondria, and 6 in the cytoplasm (Table 3).

## Discussion

Individual and combined abiotic stressors, such as drought, salinity, metal, and submergence in water, adversely affect plant growth and yield (Rizhsky et al., 2002, 2004; Mittler, 2006; Mittler and Blumwald, 2010; Atkinson and Urwin, 2012). Plant's response to the continuous exposure of CAbS conditions are more complex that are mostly influenced by diverse and antagonistic signaling pathways, which may interact and/or impede each other (Suzuki et al., 2005; Mittler and Blumwald, 2010). Meta - analysis is a way of combining the results of all the studies. It has been used to identify the genes commonly expressed under different stress conditions in recent studies (Chang et al., 2013; Shaik and Ramakrishna, 2013, 2014; Ramu et al., 2016). Here we report the transcriptomic data of *O. sativa* for eight different abiotic stresses processed using INMEX and the heatmap was derived based on the expression values of the stress responsive genes. Based on this heatmap and physical map data, 30 commonly expressed AbS responsive genes were identified and subjected to Rice DB to have an insight on the developmental tissue-specific expression of genes. Further these AbS responsive genes were chosen for expression profiling under CAbS. Public microarray hybridization of Rice DB expression values showed developmental tissue-specific expression patterns of 28 genes out of 30 AbS responsive genes based on the available transcriptomic data. The plants are affected by stress under different developmental tissues (Jin et al., 2013; Narsai et al., 2013b) and thus a large set of transcriptomic data are available to study the expression analysis leading to a better understanding of different molecular mechanisms regulating AbS (Liu, 2015). Hence these 28 genes which showed differential expression pattern in the 41 different developmental tissues showed a higher expression level of AbS responsive genes from multiple tissue and their expression during the individual or combined stresses. Thus, suggesting their multiple roles in diverse molecular and physiological activities. This data could be exploited for selecting candidate genes showing distinct expression pattern for explaining their functional roles. It highlighted the meta-analysis of genes expressed in different developmental tissues. We outlined information on co-regulation among genes under AbS conditions (Narsai et al., 2013b). By identifying each protein family involved in the stress response, the molecular mechanism of the plant stress response can be predicted. These results support the phase-specific expression of AbS responsive genes and are presumed to get expressed only during reserve deposition phase of plant tissues and seed development. This expression profiling data would facilitate the combinatorial usage of candidate genes in the transcriptional regulation of AbS related protein synthesis. Further, this supplementary data also serves as a base for conducting overexpression studies of potential genes in various plant tissues in order to increase the AbS responsive protein content in rice.

Physical mapping of 30 AbS responsive genes on the 12 chromosomes of *O. sativa* showed, that a maximum number of AbS responsive genes were present on chromosomes 5 (6 genes; ~20%) and 2 (4 genes; ~13.33%) and these physical regions contains higher accumulation of gene clusters at both upper and lower end of the arms. A minimum number of genes were present on chromosome 3, 7, 9, (3 genes; ~10% each) and 1, 4, 11, 12, (2 genes each; ~6.66% each) and a minimum of 1 gene each (~3.33%) were present on chromosomes 6, 8, and 10. It speculates lower accumulation of genes as demonstrated by Muthamilarasan et al. (2014a). It revealed the AbS responsive genes distribution of chromosomes among the genome.

Comparative mapping of AbS responsive proteins on rice, sorghum, foxtail millet and maize databases were performed to understand the orthologous connections between the grass species genomes. Rice AbS genes showed maximum synteny with maize and foxtail millet genes (~70%), followed by sorghum (~67%), however, higher percentage of orthology was expected between rice, maize and foxtail millet owing to their extensive synteny at gene level. Rice AbS responsive genes were found to be more homologous to maize and foxtail millet highlights its close evolutionary relationships while the decrease in synteny with sorghum due to the increase in genetic distance across the phylogeny (Bennetzen et al., 2012; Zhang et al., 2012). This pattern of decreasing synteny was also observed in the comparative mapping of NAC TFs (Puranik et al., 2013), WD40 genes (Mishra et al., 2014), C_2_H_2_ zinc fingers (Muthamilarasan et al., 2014a), MYB TFs (Muthamilarasan et al., 2014b), AP2/ERFs (Lata et al., 2014) and 14-3-3 proteins (Kumar et al., 2015). This comparative map data would assist in understanding the evolutionary process of AbS responsive genes among the Poaceae members.

The present report predicted certain key CAbS genes like MATH (TRAF homology)-BTB and NAC domains. MATH-BTB domain genes were found in large number at *O. sativa* and *Arabidopsis* (Gingerich et al., 2007), that are reported to be involved in expansion and diversification events in monocots and dicots (Gingerich et al., 2007). MATH-BTB domain are responsible to activate the ubiquitination that plays a candid role in the development, homeostasis, physiology, hormonal balance, cell cycle, circardian rhythms, photomorphogenesis, ecological adaptation, disease resistance, and abiotic stress tolerance (Drought and salinity) (Zapata et al., 2007; Qi et al., 2009; Zhao et al., 2013; Kushwaha et al., 2016). NAC domains (TFs) on other hand are commonly induced by CAbS that acts potential candidates to produce plants with improved CAbS tolerance (Wu et al., 2009; Shao et al., 2015). But many the time this overexpression study may lead to negative effects in rice and *Arabidosis* transgenic plants such as lower yield, late flowering, sensitive to ABA signaling and dwarfing (Nakashima et al., 2007; Hao et al., 2011; Liu et al., 2011; Lu et al., 2012; Mao et al., 2012). Therefore, to gain first insight into the key players and their potential functions of rice CAbS genes during stress response and development, we have explored publically available microarray data for rice. Further, this would also enable the researchers to select and validate the candidate genes from rice.

Commonly shared genes, their respective protein cross talks, and functional association revealed the complexity of abiotic stresses based on seed protein neighborhoods and the configuration of its nodes, connecting edges from the genes that were differentially expressed in abiotic stress studies. AbS encoding proteins and their properties revealed huge difference in the length, isoelectric point, molecular weight, instability index, aliphatic index and GRAVY values of these proteins.

## Conclusions

The emerging advancement in physiology and computational approaches has paved way to understand the role of AbS responsive genes in molecular cross talks. Gene regulation has been studied well in all the major food crops and tree species. No such study on CAbS genes has been conducted in *O. sativa*, for exploring C3 photosynthesis and AbS tolerance mechanisms. Seemingly the importance of these crop and abiotic stress genes, the current study uses well-known computational approaches to detect and describe AbS responsive genes. The identified genes were used for the construction of physical map, gene ontology annotation, comparative mapping, molecular interaction, and subcellular localization predictions. In toting, *in silico* expression profiling of rice AbS responsive (genes) transcriptomic data was performed to understand the expression pattern of these genes in different developmental tissues. These *in silico* expression analysis of 30 novel AbS responsive genes showed regulatory functions under combined stress conditions. The present study we hypothesize CAbS responsible proteins may activate various downstream gene(s), (yet to be characterized), which lead to the accumulation of osmolytes such as proline, lysine, glycine, betaine, sucrose, fructose, myo-inosital, and mannitol (these osmolytes plays important role in AbS tolerance and avoidance mechanisms) to improve the oxidative stress by scavenging chemically active ROS molecules and consequently provide CAbS endurance by altering the molecular and physiological process of the plant.

The *in silico* expression analysis infers role of CAbS in ABA dependent/ independent manner and affects regulation of ROS quenching responsible genes. Though further studies are needed to establish the exact mechanism of action of the CAbS key players in plant under diverse AbS's. In addition to the advanced technologies such as RNA-seq needs completely sequenced models are prerequisite to generate high quality transcriptomic data for understanding the regulatory mechanism of CAbS. Further validation of the current study using crops such as *A. thaliana, Setaria italica* and *O. sativa* is speculated to throw more light in depicting the function of key genes in the development of CAbS tolerance.

## Author contributions

MR and PM conceived and designed the experiments. PM, SRK, and RP performed the experiments. PM analyzed the results and wrote the manuscript. MR approved the final version of the manuscript.

### Conflict of interest statement

The authors declare that the research was conducted in the absence of any commercial or financial relationships that could be construed as a potential conflict of interest.

## Supplementary material

Supplementary Table 1 **Biological processes and molecular functions of unique and CAbS proteins**.

Supplementary Table 2 **Functional details of AbS responsive genes**.

Supplementary Table 3 **OsAffx.4454.1.S1_at (OS05G0350900) probe set with their normalized expression intensitiesand custom heatmap in MS excel**.

Supplementary Table 4 **Os.27139.2.S1_at (OS02G0612700) probe set with their normalized expression intensitiesand custom heatmap in MS excel**.

Supplementary Table 5 **OsAffx.4221.1.S1_x_at (OS05G0104200) probe set with their normalized expression intensitiesand custom heatmap in MS excel**.

Supplementary Table 6 **Os.56311.1.S1_at (OS03G0596200) probe set with their normalized expression intensitiesand custom heatmap in MS excel**.

Supplementary Table 7 **OsAffx.7567.1.S1_at (OS12G0225900) probe set with their normalized expression intensities and custom heatmap in MS excel**.

Supplementary Table 8 **OsAffx.16106.1.S1_at (OS07G0152000) probe set with their normalized expression intensities and custom heatmap in MS excel**.

Supplementary Table 9 **OsAffx.5684.1.S1_at (OS08G0119500) probe set with their normalized expression intensities and custom heatmap in MS excel**.

Supplementary Table 10 **OsAffx.28006.1.S1_at (OS06G0594700) probe set with their normalized expression intensities and custom heatmap in MS excel**.

Supplementary Table 11 **Os.18337.2.S1_x_at (OS01G0393100) probe set with their normalized expression intensities and custom heatmap in MS excel**.

Supplementary Table 12 **Orthologous relationships between *O. sativa* and *Sorghum bicolor***.

Supplementary Table 13 **Orthologous relationships between *O. sativa* and *S. italica***.

Supplementary Table 14 **Orthologous relationships between *O. sativa* and *Zea mays***.
