# Supplementary material for: Global Transcriptome Analysis of Combined Abiotic Stress Signaling Genes Unravels Key Players in Oryza sativa L.: An In silico Approach
Source: Front Plant Sci. 2017 May 15;8:759. doi: 10.3389/fpls.2017.00759 (PMC5430072; doi:10.3389/fpls.2017.00759)
Supplement: Supplementary Table 1 — Biological processes and molecular functions of unique and CAbS proteins. [file Table1.DOCX]

| **S. No** | **Gene IDs** | **Molecular Function** | **Biological process** |
| --- | --- | --- | --- |
| 1 | **OS04G0433000** | Ion binding;  Ligase activity | Response to stress  Cellular protein modification process |
| 2 | **OS02G0695050** | Ion binding ;  Ligase activity | Response to stress  Cellular protein modification process |
| 3 | **OS05G0215183** | Ion binding ;  Ligase activity | Response to stress; catabolic process  Cellular protein modification process |
| 4 | **OS09G0403300** | Oxidoreductase activity; Ion binding | Biosynthetic process; metabolic process; response to stress |
| 5 | **OS02G0604800** | Protein binding; kinase activity; enzyme regulator activity | Cell differentiation; anatomical structure development; response to stress; signal transduction |
| 6 | **OS05G0390000** | Transcription regulator activity | Metabolic process;carbohydrate metabolic process; Biosynthetic process |
| 7 | **OS01G0220900** | Ion binding ;  Ligase activity; protein binding | Response to stress ; Biosynthetic process; metabolic process |
| 8 | **OS05G0350900** | Transcription regulator activity; DNA binding | Response to stress; signal transduction; biosynthetic process; anatomical structure development; cell differentiation; cellular nitrogen compound metabolic process |
| 9 | **OS02G0612700** | DNA binding | Cellular modification process; response to stress; signal transduction |
| 10 | **OS09G0543400** | Enzyme regulator activity | Amino acid metabolic process; anatomical structure development; response to stress |
| 11 | **OS11G0650600** | Transcription regulator activity; oxidoreductase activity | Response to stress ; Biosynthetic process; metabolic process |
| 12 | **OS07G0600300** | DNA binding | Response to stress; small molecule metabolic process; cofactor metabolic process |
| 13 | **OS05G0353600** | Oxidoreductase activity | Response to stress; signal transduction; biosynthetic process; anatomical structure development; cell differentiation |
| 14 | **OS03G0179100** | Ion binding; oxidoreductase activity | Response to stress; signal transduction |
| 15 | **OS03G0278566** | Ion binding; transcription regulator activity | Response to stress; signal transduction |
| 16 | **OS02G0174400** | Kinase activity; ion binding; transcription regulator activity; protein binding | Signal transduction; response to stress; anatomical structure development; cellular protein modification process |
| 17 | **OS04G0255600** | Oxidoreductase activity; ion binding | Secondary metabolic process; cellular nitrogen compound metabolic process; response to stress; biosynthetic process; anatomical structure development; catabolic process |
| 18 | **OS03G0596200** | Ion binding; transcription regulator activity | Response to stress; biosynthetic process; anatomical structure development |
| 19 | **OS12G0225900** | Oxidoreductase activity; ion binding; transcription regulator activity | Response to stress; biosynthetic process; catabolic process; cellular nitrogen compound metabolic process |
| 20 | **OS07G0152000** | DNA binding; transcription regulator activity | Biosynthetic process; response to stress; anatomical structure development; cell differentiation |
| 21 | **OS08G0119500** | Oxidoreductase activity; ion binding | Biosynthetic process; catabolic process; cellular nitrogen compound metabolic process |
| 22 | **OS05G0360900** | Enzyme regulator activity | Response to stress; biosynthetic process; anatomical structure development |
| 23 | **OS11G0605200** | DNA binding; protein binding | Biosynthetic process; signal transduction |
| 24 | **OS12G0572700** | Transcription regulator activity; ion binding | Carbohydrate metabolic process; cellular modification process; signal transduction; anatomical structure development |
| 25 | **OS06G0594700** | Transcription regulator activity; ion binding | Biosynthetic process; Carbohydrate metabolic process; carbohydrate metabolic process; response to stress; anatomical structure development |
| 26 | **OS01G0393100** | Transcription regulator activity; DNA binding | Response to stress; anatomical structure development; signal transduction; cellular nitrogen compound metabolic process |
| 27 | **OS10G0211900** | Ion binding; protein binding; transcription regulator activity; kinase activity | Cellular protein modification process; signal transduction; anatomical structure development; cell differentiation |
| 28 | **OS07G0244800** | Transcription regulator activity | Response to stress; anatomical structure development; signal transduction |
| 29 | **OS09G0375300** | Enzyme regulator activity | Response to stress; cellular nitrogen compound metabolic process |
| 30 | **OS03G0278566** | Transcription regulator activity | Biosynthetic process; response to stress; anatomical structure development; cell differentiation |
